# Koumine enhances spinal cord 3α-hydroxysteroid oxidoreductase expression and activity in a rat model of neuropathic pain

**DOI:** 10.1186/s12990-015-0050-1

**Published:** 2015-08-09

**Authors:** Hong-Qiang Qiu, Ying Xu, Gui-Lin Jin, Jian Yang, Ming Liu, Su-Ping Li, Chang-Xi Yu

**Affiliations:** Department of Pharmacology, College of Pharmacy, Fujian Medical University, 350108 Fuzhou, Fujian People’s Republic of China; Fujian Key Laboratory of Natural Medicine Pharmacology, College of Pharmacy, Fujian Medical University, Fuzhou, Fujian People’s Republic of China

**Keywords:** Koumine, Neuropathic pain, Neurosteroids, Allopregnanolone, 3α-Hydroxysteroid dehydrogenase

## Abstract

**Background:**

Koumine is an alkaloid monomer found abundantly in *Gelsemium* plants. It has been shown to reverse thermal hyperalgesia and mechanical allodynia induced by sciatic nerve chronic constriction injury (CCI) in rats in a dose-dependent manner. Interestingly, this effect is mediated by elevated allopregnanolone levels in the spinal cord (SC). Since 3α-hydroxysteroid oxidoreductase (3α-HSOR), the key synthetase of allopregnanolone, is responsible for allopregnanolone upregulation in the SC, the objective of the present study was to investigate the role of its expression in the SC in koumine-induced analgesia using a rat model of neuropathic pain following peripheral nerve injury.

**Results:**

Time-course investigations of immunohistochemistry and real-time polymerase chain reaction revealed that the immunoreactivity and mRNA expression of 3α-HSOR markedly increased in a time-dependent manner in the SC of koumine-treated CCI rats. Furthermore, 3α-HSOR activity in the SC of koumine-treated CCI rats increased by 15.8% compared to the activity in untreated CCI rats. Intrathecal injection of medroxyprogesterone acetate, a selective 3α-HSOR inhibitor, reversed the analgesic effect of koumine on CCI-induced mechanical pain perception. Our results confirm that koumine alleviates neuropathic pain in rats with CCI by enhancing 3α-HSOR mRNA expression and bioactivity in the SC.

**Conclusion:**

This study demonstrates that 3α-HSOR is an important molecular target of koumine for alleviating neuropathic pain. Koumine may prove a promising compound for the development of novel analgesic agents effective against intractable neuropathic pain.

## Background

Neuropathic pain is pain resulting from an injury or disease of the somatosensory system [1]. A wide variety of insults to the peripheral and central nervous systems, including cerebrovascular accident, chemotherapy, nutritional deficiencies, surgery, systemic diseases, and trauma, can result in neuropathic pain. Neuropathic pain can cause abnormal pain sensations, including allodynia, hyperalgesia, dysesthesia, and spontaneous pain, which are difficult to treat. Current pharmacologic therapy for neuropathic pain consists mainly of nonsteroidal anti-inflammatory drugs (NSAIDs), opioid analgesics, anticonvulsants, antidepressants, and topical remedies. Unfortunately, the treatments available for neuropathic pain are far from satisfactory: nearly two-thirds of patients experiencing neuropathic pain receive insufficient relief [2]. Therefore, novel analgesics may contribute to the development of effective treatment strategies against neuropathic pain.

**Fig. 1:**
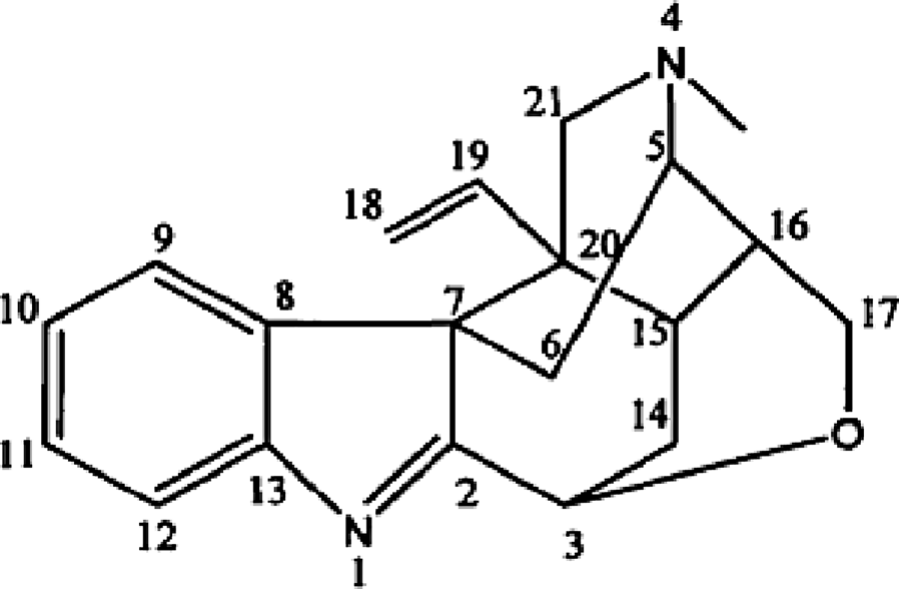
Chemical structure of koumine. The chemical structure of koumine. Molecular formula, C_20_H_22_N_2_O; molecular weight, 306.40; CAS registry number, 1358-76-5.

*Gelsemium* is a genus of the family Loganiaceae; it comprises 3 species: (1) *Gelsemium elegans* Benth. (Fig. 1), native to Asia; (2) *Gelsemium sempervirens* Ait.; and (3) *Gelsemium rankinii* Small., native to North America [3, 4]. An increasing body of evidence indicates that alkaloidal extracts from *G. elegans* Benth. elicit numerous biological effects, including analgesic, antidepressant, anxiolytic, and antitumor effects [5–9]. *G. elegans* Benth. has long been used in Chinese folk medicine to alleviate pain, inflammation, and cancer [9]. Consistently, alkaloids of *G. elegans* Benth. are thought to have analgesic properties and exhibit pharmaceutical potential [10, 11]. The most abundant alkaloid in *G. elegans* Benth. is koumine (molecular formula, C_20_H_22_N_2_O; molecular weight, 306.30; CAS registry number, 1358-76-5) (Fig. 1). According to our previous behavioral observations in animals, koumine reverses chronic constriction injury (CCI) to the sciatic nerve and thermal hyperalgesia induced by lumbar 5 (L5) spinal nerve ligation (SNL) in a dose-dependent manner. Furthermore, mechanical allodynia in rats is reduced by koumine in a dose-dependent manner [12]. Koumine differs substantially from the currently available analgesics, since it belongs to a class of chemicals known as indole alkaloids. Moreover, it lacks the adverse effects associated with most analgesic agents [6, 11]. Therefore, we hypothesized that the analgesic profile and underlying mechanism by which koumine induces analgesia are unique.

Allopregnanolone, also known as 3α, 5α-tetrahydroprogesterone (3α, 5α-THP), is one of the most important neuroactive steroids. Upregulation of allopregnanolone was shown to induce significant analgesia, implying that allopregnanolone in the spinal cord (SC) may be an important key modulator of neuropathic pain. Interestingly, our previous work has demonstrated that increased allopregnanolone levels in the SC mediated the analgesic effect of koumine on neuropathic pain [12]. Although allopregnanolone has been found to be upregulated in the SC of rats with CCI following koumine treatment, little is known about the cellular and molecular mechanisms underlying its antinociceptive actions. Since allopregnanolone biosynthesis is dependent on the activity of 3α-hydroxysteroid oxidoreductase (3α-HSOR), we performed molecular time-course experiments to analyze 3α-HSOR’s cellular distribution, gene expression, and bioactivity in the lumbar SC following koumine treatment of CCI-induced pain symptoms. The aim of this study was to investigate the relationship between the analgesic effect of koumine on neuropathic pain and 3α-HSOR in SC after peripheral nerve injury in rats to clarify koumine’s analgesic mechanism of action.

## Results

### The effect of koumine on CCI-induced neuropathic pain in rats

We have previously demonstrated that koumine has no effects in sham CCI rats [12]. In the current study, two-way repeated measures ANOVA of the thermal withdrawal latency (TWL) and mechanical withdrawal threshold (MWT) measurement values of the hind paw ipsilateral to the CCI demonstrated a significant treatment effect between subjects (*F*_5,210_ = 1,463.57, *P* < 0.001 for TWL and *F*_5,210_ = 167.03, *P* < 0.001 for MWT) and treatment time (*F*_6,210_ = 1,816.41, *P* < 0.001 for TWL and *F*_6,210_ = 451.51, *P* < 0.001 for MWT). Furthermore, a significant interaction was found between treatment and time (*F*_30,210_ = 171.84, *P* < 0.001 for TWL and *F*_30,210_ = 36.35, *P* < 0.001 for MWT). Analysis with post hoc Dunnett’s T3 tests indicated that CCI significantly decreased the thermal withdrawal latency to thermal stimulation (*P* < 0.001, vs. the sham group) and the mechanical withdrawal threshold to mechanical stimulation (*P* < 0.001, vs. the sham group). These findings demonstrated that the development of thermal hyperalgesia and mechanical allodynia peaked on postoperative day 8 and 10, respectively. Furthermore, both conditions persisted for the entire observation period.

**Fig. 2:**
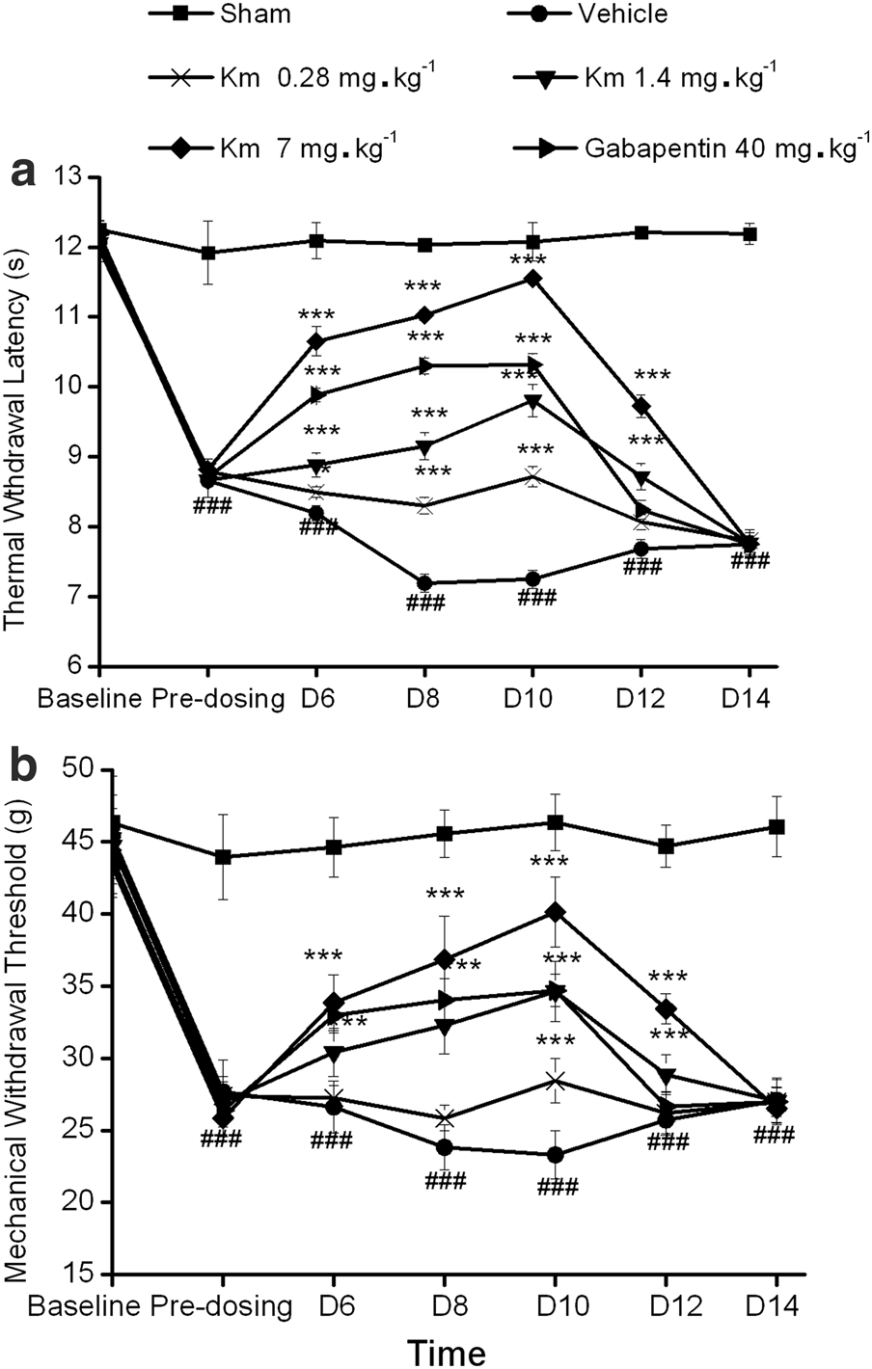
The effects of a repeated subcutaneous administration of koumine in sciatic nerve chronic constriction injury rats. Koumine (0.28, 1.4, or 7 mg kg^−1^ body weight (bw)), gabapentin (40 mg kg^−1^ bw), or vehicle was administered twice daily for 7 consecutive days, starting on postoperative day 4. The time course of the effect of koumine on the thermal withdrawal latency (TWL, **a**) and mechanical withdrawal threshold (MWT, **b**) revealed that repeated subcutaneous (s.c.) injections of koumine dose-dependently reversed hyperalgesia and allodynia induced by sciatic nerve chronic constriction injury (CCI) neuropathy. The data are presented as the mean ± SEM (n = 6 per group) and were analyzed using two-way repeated measures ANOVA. The significant differences between the groups were determined by Bonferroni post hoc test at each time point. ^###^ *P* < 0.001 vs. the sham group; *P < 0.05, **P < 0.01, ***P < 0.001 vs. the vehicle control group.

In these experiments, gabapentin [40 mg kg^−1^ body weight (bw)] significantly attenuated thermal hyperalgesia (*P* < 0.001) and mechanical allodynia (*P* < 0.05) compared to the effect of the vehicle. Twice-daily subcutaneous (s.c.) administration of koumine (7 mg kg^−1^ bw) between postoperative day 4 and 10 also significantly reversed thermal hyperalgesia (*P* < 0.001) and mechanical allodynia (*P* < 0.01) compared to the effect of the vehicle. Interestingly, koumine exhibited more potent suppression of thermal hyperalgesia (*P* < 0.01) and mechanical allodynia (*P* < 0.01) than was observed for gabapentin. Koumine exerted dose-dependent (two-way repeated measures ANOVA, *F*_2,105_ = 290.07, *P* < 0.001 for TWL and *F*_2,105_ = 424.15, *P* < 0.001 for MWT) and time-dependent (two-way repeated measures ANOVA, *F*_6,105_ = 1,666.7, *P* < 0.001 for TWL and *F*_2,105_ = 22.34, *P* < 0.001 for MWT) analgesic effects. As shown in Fig. 2, koumine administration (7 mg kg^−1^ bw, s.c.) induced analgesia within 2 days. The maximum analgesic effect was reached on day 7 of treatment, and was maintained for 2 days after koumine withdrawal. Together, these findings suggest that koumine may reverse neuropathic pain.

### The effect of koumine on 3α-HSOR immunoreactivity in the dorsal horn of L5–L6 SC of rats with CCI neuropathy

**Fig. 3:**
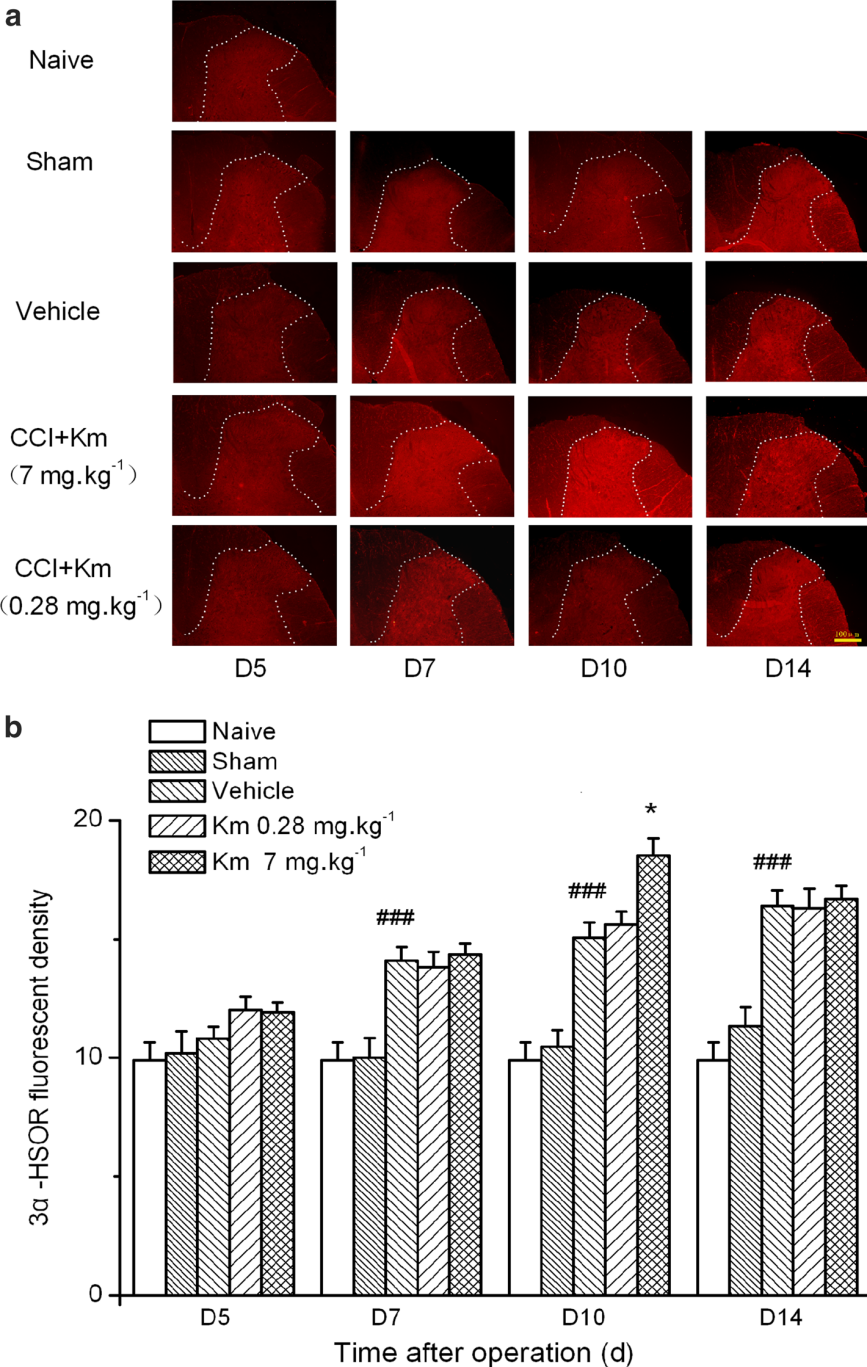
3α-Hydroxysteroid oxidoreductase immunohistochemical staining in the spinal cord of koumine-treated sciatic nerve chronic constriction injury rats. **a** 3α-Hydroxysteroid oxidoreductase (3α-HSOR) immunohistochemical staining in the ipsilateral dorsal horn of the lumbar spinal cord (SC, L5–L6). *Scale bar* 100 μm. **b** Quantification of the 3α-HSOR expression in the SC after chronic constriction injury (CCI) by fluorescence density analysis. A time-dependent increase in 3α-HSOR fluorescence density was observed within the ipsilateral SC dorsal horn after CCI. The data are presented as the means ± SEM from 5 to 7 rats per group and were analyzed using two-way ANOVA followed by Bonferroni post hoc test at each time point. ^##^ *P* < 0.01 vs. the sham group; *P < 0.05 vs. the CCI group.

**Fig. 4:**
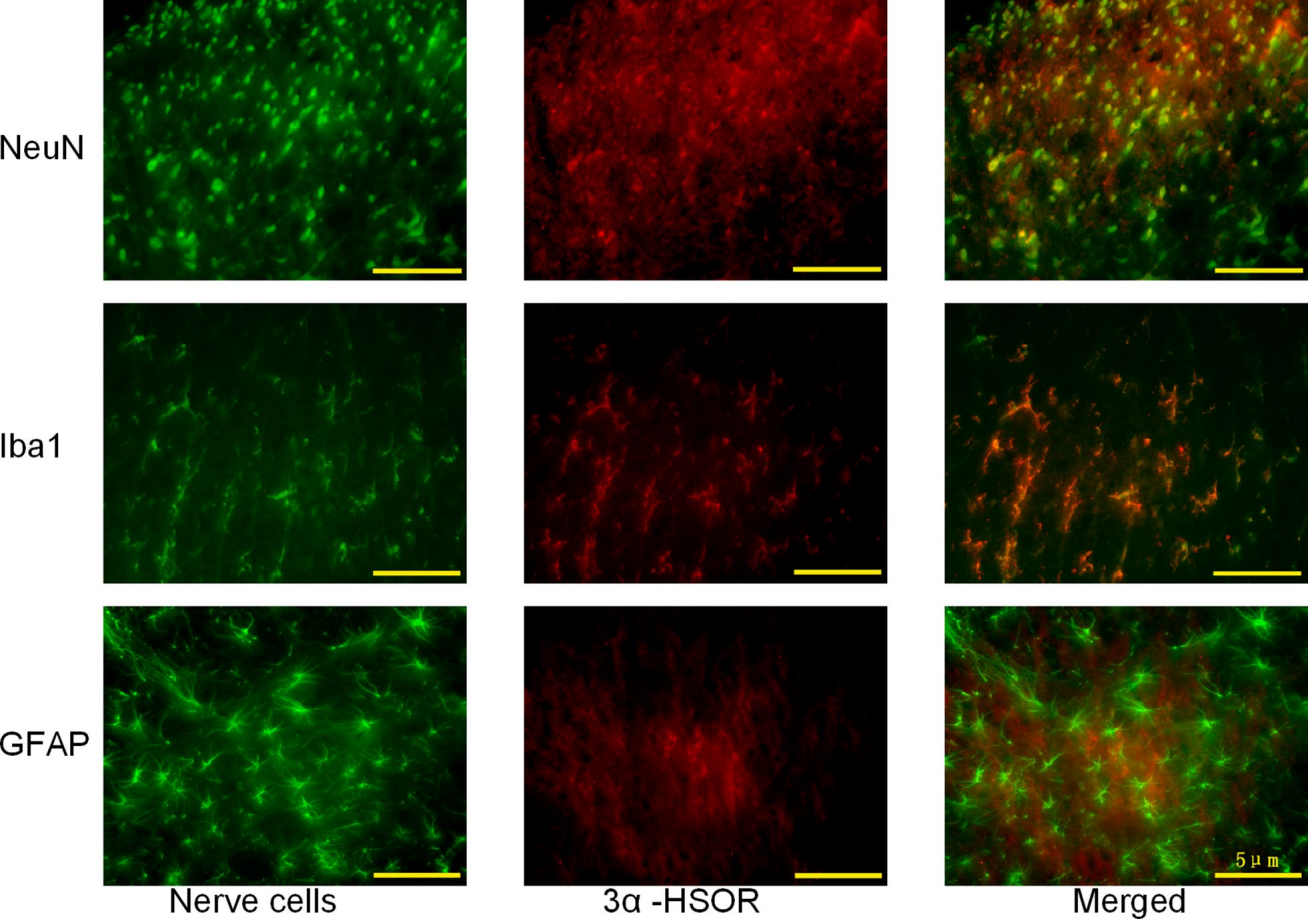
Immunostaining of the nerve cellular distribution of 3α-hydroxysteroid oxidoreductase in the dorsal horn of rat spinal cord. *Left* (nerve cells): Photomicrograph of the dorsal horn section labeled with anti-neuronal nuclei (NeuN), anti-ionized calcium binding adaptor molecule 1 (Iba1), and anti-glial fibrillary acidic protein (GFAP) antibody (*green*). Center (3α-HSOR): The same section was labeled with anti-3α-hydroxysteroid oxidoreductase (3α-HSOR) antibody (*red*). *Right* (merged): Photomicrograph of the same section labeled with anti-3α-HSOR antibody and either anti-NeuN, anti-Iba1, or anti-GFAP antibody. *Scale bar* 5 μm.

We previously determined that elevated levels of allopregnanolone, a neurosteroid present in the SC, mediated the analgesic effect of koumine [12]. To further investigate the exact mechanism of allopregnanolone upregulation, the spinal distribution of 3α-HSOR, the key synthetase of allopregnanolone, was assessed by fluorescence immunohistochemistry in koumine-treated CCI rats. The immunoreactivity of 3α-HSOR in the dorsal horn of L5–L6 SC ipsilateral to CCI is shown in Fig. 3a. A two-way ANOVA performed on the 3α-HSOR fluorescence density data revealed a significant treatment effect between subjects (*F*_4,100_ = 61.21, *P* < 0.001) and that 3α-HSOR fluorescence density significantly changed with treatment time (*F*_3,100_ = 11.35, *P* < 0.001). Furthermore, there was a significant interaction between treatment and time (*F*_12,100_ = 3.53, *P* < 0.001). Bonferroni post hoc test revealed that CCI treatment significantly increased 3α-HSOR immunofluorescence staining density (*P* < 0.001 vs. the sham group) from postoperative day 7 to the end of the observation period. In contrast to the findings in the CCI group, the treatment- and surgery-naïve (“naïve”) and sham groups showed no changes in 3α-HSOR immunofluorescence density during the entire observation period. Twice-daily administration of koumine (7 mg kg^−1^ bw s.c.) between day 4 and 10 further increased 3α-HSOR immunofluorescence staining density, reaching a maximum after 7 consecutive days of treatment (*P* < 0.001 vs. the vehicle group). As shown in Fig. 3b, no differences in dorsal horn 3α-HSOR immunofluorescence density were observed between the CCI and koumine-treated group on postoperative day 14 (4 days after koumine withdrawal). Using immunofluorescence double labeling we found that 3α-HSOR was widely distributed in the dorsal horn of the SC, and was co-expressed mainly with neurons and microglia (Fig. 4).

### The effect of koumine on *3α*-*HSOR* mRNA expression in the dorsal horn of rat L5–L6 SC after CCI-induced neuropathic pain

**Fig. 5:**
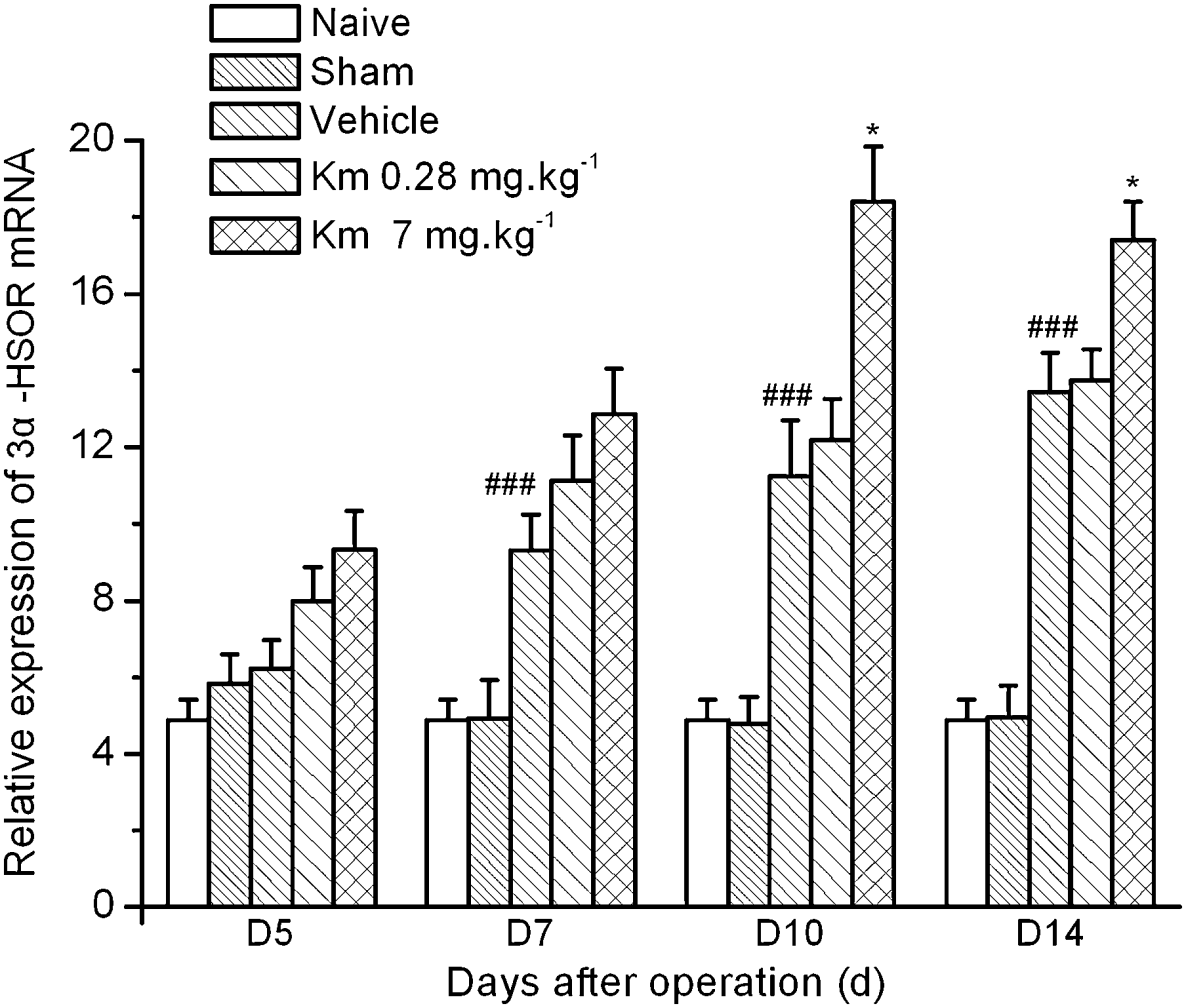
The effect of koumine on *3α*-*hydroxysteroid oxidoreductase* mRNA expression in the spinal cord of sciatic nerve chronic constriction injury rats. The *3α*-*hydroxysteroid oxidoreductase* (*3α*-*HSOR*) mRNA level is expressed as the ratio of *glyceraldehyde*-*3*-*phosphate dehydrogenase* (*GAPDH*) mRNA in the spinal cord (SC) of naïve, sham-operated, sciatic nerve chronic constriction injury (CCI), or koumine-treated rats. The data are presented as the mean ± SEM (n = 6 per group) and were analyzed by two-way ANOVA followed by Bonferroni post hoc test at each time point. ^##^ *P* < 0.01 vs. the sham group; *P < 0.05 vs. the CCI group.

Since 3α-HSOR immunostaining in the dorsal horn of the SC of CCI rats was increased after koumine administration, we determined lumbar *3α*-*HSOR* mRNA expression by reverse transcription polymerase chain reaction (RT-PCR) in the same koumine-treated CCI rats. Spinal RNA was extracted and its integrality and concentration were determined. A two-way ANOVA performed on *3α*-*HSOR* mRNA expression in the SC ipsilateral to CCI also demonstrated a significant treatment effect between subjects (*F*_4,100_ = 75.89, *P* < 0.001) and treatment time (*F*_3,100_ = 18.34, *P* < 0.001), and a significant interaction between treatment and time (*F*_12,100_ = 4.72, *P* < 0.001) (Fig. 5). In agreement with the results of the immunochemistry analysis, Bonferroni post hoc test showed that CCI significantly enhanced *3α*-*HSOR* mRNA expression (*P* < 0.001 vs. sham group) from postoperative day 7 to the end of the observation period. In contrast, the naïve and sham groups showed no difference in *3α*-*HSOR* mRNA expression during the observation period (*P* > 0.05, vs. the CCI group). Twice-daily administration of koumine (7 mg kg^−1^ bw, s.c.) between postoperative day 4 and day 10 further increased *3α*-*HSOR* mRNA expression and reached a maximum after 7 consecutive days of treatment (*P* < 0.05 vs. the CCI group). However, as shown in Fig. 5, compared to the *3α*-*HSOR* mRNA expression in the CCI group, koumine-treated rats demonstrated a noticeable *3α*-*HSOR* mRNA expression upregulation on postoperative day 14, i.e., 4 days after koumine withdrawal (*P* < 0.05).

### The effect of koumine on 3α-HSOR catalytic activity in rat SC after CCI-induced neuropathic pain

**Fig. 6:**
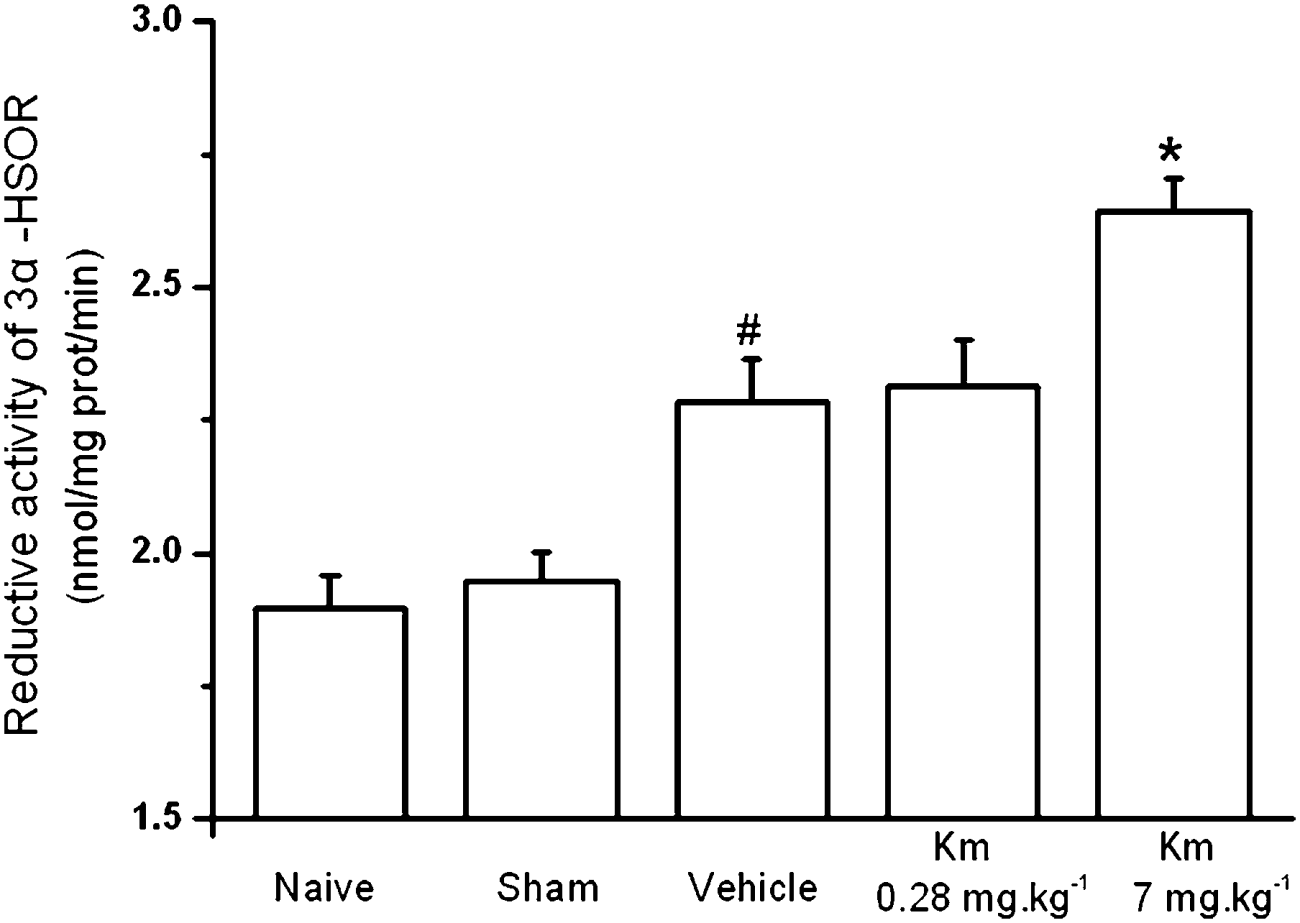
The effect of koumine on 3α-hydroxysteroid oxidoreductase catalytic activity in the spinal cord of sciatic nerve chronic constriction injury rats. The 3α-hydroxysteroid oxidoreductase **(**3*α*-HSOR) catalytic activity in the spinal cord (SC) lumbar region L5–L6 was assessed spectrophotometrically by measuring the oxidation rate of nicotinamide adenine dinucleotide phosphate (NADPH) at 340 nm and 37°C. The data are presented as the mean ± SEM (n = 6 per group) and were analyzed by one-way ANOVA followed by Bonferroni post hoc test at each time point. *P < 0.05 vs. the CCI group, ^#^ *P* < 0.05 vs. the sham group.

The effect of koumine on spinal 3α-HSOR activity in CCI rats was determined by enzyme kinetics analysis. A significant treatment effect on the 3α-HSOR catalytic activity was observed between the rats (one-way ANOVA, *F*_4,25_ = 19.19, *P* < 0.001). As shown in Fig. 6, 3α-HSOR activity was significantly increased by 17.4% in CCI rats (*P* < 0.05 vs. the sham group). After 7 consecutive days of koumine administration (7 mg kg^−1^ bw, s.c.), 3α-HSOR activity in the SC of CCI rats was further enhanced by 15.8% (*P* < 0.05 vs. the CCI group). This finding implies that the increased 3α-HSOR mRNA expression and immunostaining in the SC of koumine-treated CCI rats may enhance 3α-HSOR bioactivity and upregulate allopregnanolone in the SC.

### The effect of medroxyprogesterone acetate on the analgesic effect of koumine in mechanical allodynia tests in CCI rats

**Fig. 7:**
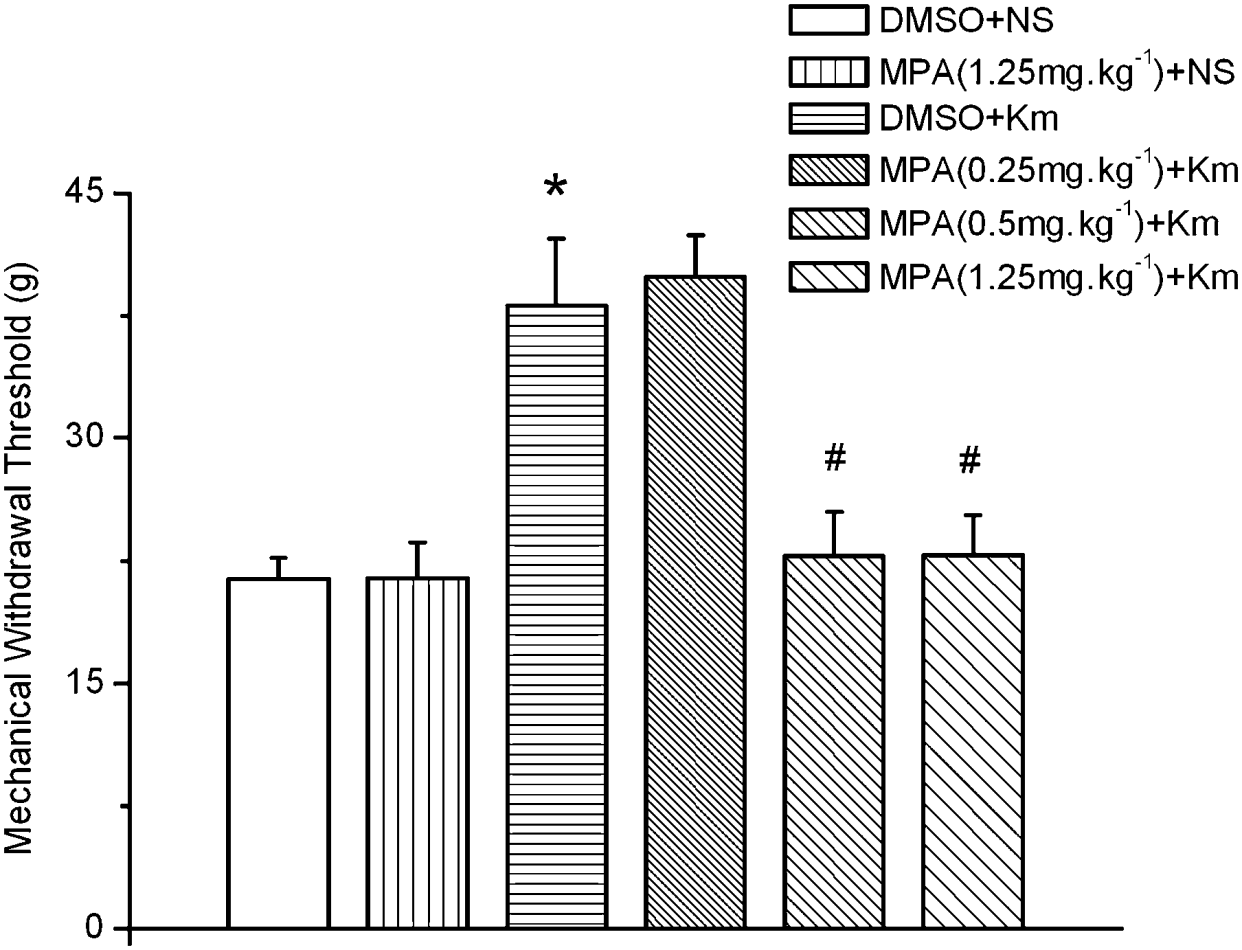
The analgesic effect of koumine was reversed in sciatic nerve chronic constriction injury rats by intrathecal treatment with medroxyprogesterone acetate. Medroxyprogesterone acetate (MPA) or dimethyl sulfoxide (DMSO, vehicle) was administered via an intrathecal catheter 10 days after sciatic nerve chronic constriction injury (CCI) surgery. After 30 min, koumine (7 mg kg^−1^ bw) or normal saline (NS) was administered by subcutaneous (s.c.) injection. The mechanical withdrawal threshold (MWT) of the hind paws was measured 1 h after completion of the drug or vehicle administration. The data are presented as the mean ± SEM (n = 6 per group) and were analyzed by one-way ANOVA followed by Bonferroni post hoc test at each time point. *P < 0.05 vs. the DMSO + NS group; ^#^ *P* < 0.05 vs. the DMSO + koumine group.

We used the 3α-HSOR inhibitor medroxyprogesterone acetate (MPA, administered by intrathecal injection) to confirm the hypothesis that the enhanced expression and activity of 3α-HSOR, which increase allopregnanolone levels in the SC of koumine-treated CCI rats, mediates analgesia. As shown in Fig. 7, the MWT in the hind paw ipsilateral to the CCI revealed a significant inter-group treatment effect (*F*_5,30_ = 9.496, *P* < 0.001, one-way ANOVA). Furthermore, s.c.-injected koumine significantly relieved mechanical allodynia compared to the effect of the vehicle (dimethyl sulfoxide + normal saline, DMSO + NS) group (*P* < 0.05). Conversely, intrathecal injection of MPA dose-dependently reversed the analgesic effect of koumine (*F*_2,15_ = 14.511, *P* < 0.001). MPA (0.5 and 1.25 mg kg^−1^ bw) significantly reduced the MWT (*P* < 0.001 vs. the DMSO + koumine group). However, the highest dose of MPA tested (1.25 mg kg^−1^ bw) had no effect on the MWT of untreated CCI rats (*P* > 0.05 vs. the DMSO + NS group). These data suggest that the analgesic effect of koumine may be linked to increased 3α-HSOR activity in the SC.

## Discussion

*Gelsemium elegans* Benth. has diverse biological effects; however, its clinical use is hampered by its toxicity. The crude alkaloid *Gelsemium* extract exhibits a high level of toxicity with an LD_50_ of 15 and 4 mg kg^−1^ bw following intragastric and intraperitoneal (i.p.) administration, respectively, in rats [13]. Several research groups are currently trying to derive alkaloid monomers with high potency and low toxicity from *G. elegans* Benth. In a previous study, we were able to obtain several different alkaloid monomers using the pH-zone–refining counter-current chromatography technique [14], which enabled us to perform pharmacodynamic screening. Our preliminary findings suggested that the most toxic alkaloid derived from *G. elegans* Benth., gelsenicine, exerted analgesic activity during inflammatory and neuropathic pain [5]. Recently, Zhang et al. reported that gelsemine, another *G. elegans* Benth.-derived main alkaloid, displayed potent and specific antinociceptive properties in chronic pain [15]. Furthermore, we previously demonstrated that koumine exhibited potent analgesic effects with a relatively low toxicity compared to that of other *G. elegans* Benth. alkaloid extracts [12, 16]. Koumine’s acute toxicity was previously investigated in both mice and rats. The LD_50_ of s.c.-administered koumine to mice was 99.0 mg kg^−1^ bw [12]. Acute lethality generally occurred within 10 min of s.c. injection in mice, with labored respiration and brief coordinated clonic convulsions occurring immediately before death [12]. Interestingly, koumine’s toxicity is much lower than that of the crude *G. elegans* extract. The LD_50_ of intragastrically administered koumine was 300 mg kg^−1^ bw in rats (unpublished data), a level much higher than that estimated for the crude alkaloidal extract (15 mg kg^−1^ bw) using the same administration route [13]. Moreover, in a previous study, the spontaneous travel velocity was not affected after administration of koumine in mice as determined by the spontaneous motor activity test [17], and no adverse effects were observed in the rats treated with a range of koumine doses (0.28–7 mg kg^−1^ bw) in the current study. The relatively wide therapeutic index of koumine suggests that it may be a promising agent in clinical applications. In this study, the TWL and MWT behavioral data indicated that koumine may ameliorate sciatic nerve CCI-induced chronic pain. Repeated administration of koumine reversed thermal hyperalgesia and mechanical allodynia in a dose-dependent manner in our CCI model. Additionally, our previous work has provided evidence of koumine’s analgesic effect in a number of animal models of pain, including SNL and diabetic-induced neuropathic pain [12, 18], indicating a role for koumine in the treatment of peripheral neuropathic pain.

Repeated s.c. administration of koumine was not associated with adverse effects commonly associated with opioids, such as physical and psychological dependence [19]. Interestingly, LaBuda and Little [20] revealed that agents such as gabapentin and morphine completely or partially reversed tactile allodynia in L5 SNL, another animal model of painful peripheral neuropathy. Conversely, indomethacin had no effect. Koumine also exhibited positive analgesic effects in the L5 SNL model [12]. Taken together, these findings suggest that koumine’s analgesic mechanism of action differs from that of NSAIDs and opioids. Interestingly, we found that koumine exhibited its analgesic effect by upregulating allopregnanolone, one of the most potent neurosteroids in the SC [12].

The pathophysiology underlying neuropathic pain is complicated, with many aspects remaining unclear. A striking finding in the past three decades was the discovery that neurons are capable of synthesizing neurosteroids independently of the classical mechanism involving endocrine glands [21, 22]. The involvement of endogenous neurosteroids is well established in the area of chronic pain modulation [21–25]. Inflammatory and neuropathic pain states are associated with the upregulated synthesis of endogenous neurosteroids in the SC, such as allopregnanolone and pregnenolone [21, 23, 26]. The administration of exogenous allopregnanolone has been shown to prevent and suppress oxaliplatin-evoked painful neuropathy [25]. Allopregnanolone is an allosteric modulator that controls several important neurophysiological mechanisms through its actions on the γ-aminobutyric acid A (GABA_A_) channel [27]. Indeed, neurosteroids modulate pain perception by potentiating the effects at GABA_A_ channels and inhibiting T-type calcium channels located in peripheral sensory neurons [23, 28–30]. Furthermore, neurosteroid synthesis has been reported in the SC, partly explaining the vital role of SC in the control of pain processing. The SC contains key enzymes related to steroid synthesis, including cytochrome P450 side chain cleavage enzymes, 3α-reductase, and 3α-HSOR [22, 23, 31]. Therefore, the key cellular and molecular components involved in neurosteroid biosynthesis in the SC may provide potential targets that may be useful for the development of novel analgesics for the treatment of persevering neuropathic pain. Consequently, an increasing number of scientists are focusing their research toward the discovery of novel neurosteroid-directed analgesics [29, 30, 32–34].

3α-HSOR belongs to the aldo–keto reductase superfamily. It is crucial for the synthesis of neuroactive 3α-reduced steroids, such as allopregnanolone and tetrahydrodeoxycorticosterone, in a reversible manner. The dorsal horn of the SC, a pivotal structure that controls pain and nociception, exhibits intense immunostaining of 3α-HSOR [22, 35]. Furthermore, neurons and glial cells synthesize various neurosteroids. Our double immunolabeling experiments showed 3α-HSOR immunostaining in SC neurons, microglia, and astrocytes. This prompted us to investigate the relationship between koumine and 3α-HSOR and koumine’s effects on allopregnanolone upregulation in the SC. The present study draws on data generated by different experimental techniques that clearly show that koumine increases the cellular immunoreactivity, gene expression, and bioactivity of 3α-HSOR in SC during chronic pain. Importantly, our time-course experiments revealed that the koumine-induced upregulation of 3α-HSOR cellular immunoreactivity and gene expression correlated with the development of neuropathic pain symptoms.

Consistent with these findings, our in vivo enzymatic activity assays revealed that the catalytic activity of 3α-HSOR in the SC was increased in CCI rats after 7 days of koumine treatment. Furthermore, we observed that koumine produced significant analgesia and dose-dependently reversed the mechanical pain thresholds induced by the intrathecal injection of the 3α-HSOR pharmacological inhibitor MPA at a dose of 0.5 or 1.25 mg kg^−1^ bw. It is worth noting that the highest dose of MPA used (1.25 mg kg^−1^ bw) had no effect on the MWT values measured in naïve CCI rats. Thus, inhibiting 3α-HSOR activity in the sensory nerve circuit of the SC could reverse the analgesic effect of koumine in CCI rats. Our findings demonstrate the direct role of 3α-HSOR in mediating pain modulation and koumine’s influence thereon. Therefore, 3α-HSOR may be a fundamental molecular target for koumine in the modulation of pain sensation. However, although the 3α-HSOR fluorescence density and mRNA expression were still elevated on postoperative day 14, the analgesic effect had disappeared by then (Figs. 3, 5). One possibility is that koumine may regulate the catalytic activity of 3α-HSOR. Allopregnanolone biosynthesis in the SC may thus be insufficient in the absence of koumine because of low 3α-HSOR catalytic activity. However, this hypothesis needs to be confirmed by the determination of allopregnanolone levels in the SC on postoperative day 14. Another explanation may be that a molecular target other than 3α-HSOR, and whose function is also altered by koumine, participates in the observed analgesic effect of koumine.

The endogenous biosynthesis of neurosteroids is also upregulated in the SC during inflammatory and neuropathic pain states [21, 36, 37]. We demonstrated a similar increase in allopregnanolone and pregnenolone levels in CCI rats [12]. The observed increase in allopregnanolone levels in CCI rats is consistent with the findings of Kawano et al. in SNL neuropathy [37]. Similarly, we found increased immunohistochemical staining, mRNA expression, and bioactivity of 3α-HSOR in the SC during sciatic nerve CCI-induced chronic pain. On the basis of the results of our and other studies, we consider that a state of chronic pain in animals is predominantly determined by two sets of factors. The first includes the pro-nociceptive mechanisms mediated by neurotransmitters supporting the development and maintenance of pain symptoms, such as substance P, bradykinin, prostaglandin, and histamine. The second group of factors opposes the pro-nociceptive processes, and its effects are mediated by endorphins, neurosteroids, and neuroprotective factors that possess adaptive or antinociceptive properties. The latter help the animal to cope with or accelerate the recovery from the pathological pain state. Consequently, the selective upregulation of the 3α-HSOR immunoreactivity, mRNA expression, and bioactivity and the 3α-HSOR-induced increase in spinal synthesis of endogenous allopregnanolone may represent an intrinsic adaptive response to neuropathic pain and elicit beneficial effects against diverse pathological pain symptoms. However, these natural or adaptive mechanisms are not sufficient to achieve the suppression of pain sensation in rats with CCI [21]. As observed in rats with CCI, an insufficient increase in 3α-HSOR and allopregnanolone levels fails to reverse the development of neuropathic pain. Only a sufficient increase in allopregnanolone would offer adequate protection against the development of neuropathic pain [12, 37]. Allopregnanolone that is endogenously formed in the central nervous system significantly alters nociception through paracrine and autocrine mechanisms [36, 38]. Therefore, koumine and other alkaloid extracts of *G. elegans* Benth. that are capable of stimulating allopregnanolone formation in neural networks may provide a novel approach for the development of analgesic therapies [39].

Our fluorescence immunohistochemistry experiments also revealed that 3α-HSOR was widely distributed in the SC dorsal horn and co-localized with neural and non-neural cells, including neurons and microglia (Fig. 4, merged photomicrograph). In recent years, there has been a growing consensus that glial cells located in the spinal dorsal horn are activated following peripheral nerve injury [40–42]. Microglial activation induces the release of proinflammatory cytokines, such as interleukin-β, tumor necrosis factor-α, and interleukin-6, which play important roles in the development of neuropathic pain. The protective property of allopregnanolone in pain perception in relation to glial cells has been well documented [25, 34, 43]. Allopregnanolone treatment not only significantly reduced astrocyte proliferation and microglial activation, but also enhanced myelination in mice [44]. Neurosteroids, such as allopregnanolone, could reduce inflammatory cytokine levels, which were, for example, elevated following traumatic brain injury [45]. Our findings demonstrated that koumine administration increased 3α-HSOR activity, which could contribute to increased allopregnanolone levels in the SC of CCI rats. Consequently, we hypothesized that elevated allopregnanolone levels may exert analgesic effects through allosteric modulation of GABA_A_ and by suppressing the release of microglia activation-induced inflammatory cytokines. Further studies are warranted to determine whether koumine influences the activation of glial cells during the neuropathic pain state.

## Conclusion

This study demonstrated that koumine could relieve neuropathic pain in rats by enhancing the mRNA expression and bioactivity of 3α-HSOR in the SC. Our study also suggested that by targeting 3α-HSOR, koumine altered 3α-HSOR-regulated allopregnanolone levels in the SC of rats. Therefore, koumine may be promising in the search for novel analgesic agents that are protective against painful neuropathy.

## Methods

### Chemicals and reagents

Koumine (99% purity) was isolated from *G. elegans* Benth. by pH-zone–refining counter-current chromatography as described previously [14]. Gabapentin (purity: 99%; Shanghai Sunheat Chemicals Co., Ltd, Shanghai, China) was used as positive control. Rabbit polyclonal antibody against 3α-HSOR was purchased from Biosynthesis Biotechnology (Beijing, China) and mouse anti-neuronal nuclei monoclonal antibody (anti-NeuN) was purchased from GeneTex (Irvine, CA, USA). Goat anti-ionized calcium binding adaptor molecule 1 antibody (anti-Iba1; catalog no. ab5076) and mouse anti-glial fibrillary acidic protein antibody (anti-GFAP; catalog no. ab4648) were purchased from Abcam (Cambridge, UK). Fluorescein (FITC)-conjugated donkey anti-mouse, tetramethylrhodamine (TRITC)-conjugated goat anti-rabbit, and Alexa Fluor^®^ 488-conjugated donkey anti-mouse were purchased from Jackson ImmunoResearch (West Grove, PA, USA). Normal rabbit and donkey serum were purchased from Biosynthesis Biotechnology (Beijing, China). PCR reagent kits, PrimeScript^®^ RT Reagent Kit, and SYBR^®^ Premix Ex Taq™ II Real-Time PCR Reagent Kit, were purchased from Takara Biotechnology Co., Ltd. (Dalian, China). Nicotinamide adenine dinucleotide phosphate-oxidase (NADPH; Roche, Pleasanton, CA, USA), 5α-dihydroprogesterone (5α-DHP; Sigma-Aldrich, St. Louis, MO, USA), medroxyprogesterone acetate (MPA; Selleck, Houston, TX, USA), and chloral hydrate (Sigma-Aldrich, St. Louis, MO, USA) were of pharmaceutical grade. All other reagents used were of analytical grade.

Koumine was prepared daily prior to use in sterile physiological saline (0.9% w/v sodium chloride), and was administered by s.c. injection at a dose of 4 mL kg^−1^ rat bw.

### Animals

Male adult Sprague–Dawley rats (180–200 g) were obtained from the Shanghai Laboratory Animal Center at the Chinese Academy of Sciences (Shanghai, China). All rat experiments were performed in accordance with the National Institutes of Health Guide for Care and Use of Laboratory Animals (Publication No. 85-23, revised 1985) and were conducted under the authority of the Committee of Ethics of the Fujian Medical University (Fujian, China). All procedures complied with the guidelines for animal care and use established at the Fujian Medical University. The rats were housed in a temperature-controlled room (25 ± 2°C) under a 12-h light/dark cycle (lights on: 08:00 AM), with access to standard laboratory food and water ad libitum, except during behavioral observations. The rats were acclimatized for at least 1 week before undergoing any experiments. Each rat was assigned to one specific behavioral experiment, and the experiments were performed between 09:00 and 17:00.

### In vivo intrathecal catheter implantation and drug administration

Intrathecal implantation of polyethylene (PE) tubing (Intramedic PE-10, Clay Adams, Parsippany, NJ, USA) into the subarachnoid space of the lumbar enlargement was performed in rats as described previously [46]. This method permits the direct administration of a drug of interest. After 1 day of recovery post surgery, the rats that were considered neurologically healthy received 2% lidocaine (20 μL) through the intrathecal catheter to confirm post-surgical placement of the PE tubing within the subarachnoid space. Those rats that displayed complete paralysis of both hind limbs and the tail following the administration of lidocaine were used for the subsequent experiments.

After recovery from intrathecal catheter placement, peripheral neuropathy was induced by CCI. The 3α-HSOR selective inhibitor MPA was used to evaluate the analgesic actions of koumine. The rats undergoing mechanical allodynia were assigned to groups receiving either DMSO (vehicle for MPA) with NS, MPA (1.25 mg kg^−1^ bw) with NS, DMSO with koumine, or MPA (0.25, 0.5, or 1.25 mg kg^−1^ bw) with koumine. On postoperative (CCI) day 10, DMSO or MPA was administered via the intrathecal catheter. After 30 min, koumine or NS was administered by s.c. injection. The MWT of the hind paws was measured 1 h after completion of the drug administration.

Visual confirmation of the placement of the PE tubing in the intrathecal space at the lumbar enlargement was performed by exposing the lumbar SC at the end of each experiment. The data obtained from rats with an incorrect PE tubing position were excluded from the study.

### Rat CCI model

The rats were prepared for the induction of CCI according to the method described by Bennett et al. [47]. The rats were anesthetized by i.p. administration of 400 mg kg^−1^ bw chloral hydrate. Subsequently, the right common sciatic nerve isolated at the level of the mid-thigh was loosely ligated using four chromic gut (5-0) ties at 1-mm intervals. The same procedure was performed without ligation in the rats assigned to the sham group. The rats were monitored for 3 days following surgery and were only used in the subsequent studies if the baseline thermal hyperalgesia and mechanical allodynia test scores (described in “Measurement of thermal hyperalgesia and mechanical allodynia in rats” below) surpassed the acceptance threshold. Baseline threshold scores were calculated as the CCI ipsilateral paw baseline score/contralateral paw baseline score. Rats displaying baseline scores between 0.8 and 1.2 were accepted into the study. Rats with thermal predose latency scores >0.8, mechanical predose threshold scores >0.75, and/or demonstrating motor deficits after surgery were excluded from the subsequent experiments.

### Measurement of thermal hyperalgesia and mechanical allodynia in rats

Thermal hyperalgesia was measured with a commercial thermal paw stimulator (PL-200, Chengdu Technology & Market Co., Ltd., Sichuan, China) as described by Hargreaves et al. [48]. The rats were placed in individual plastic cubicles mounted on a glass surface in a temperature-controlled room (25 ± 2°C). The plantar surface of each hind paw was subsequently exposed to a thermal stimulus, i.e., radiant heat emitted from a focused projection bulb, for a maximum exposure time of 16 s to minimize potential tissue damage. The second hind paw of each rat was tested after a 10-min interval. The paw TWL was calculated as the mean of the 2 hind paw withdrawal times.

CCI rats were assigned to groups receiving the vehicle, koumine (0.28, 1.4, or 7.0 mg kg^−1^ bw), or gabapentin (40 mg kg^−1^ bw) twice daily by s.c. injection for 7 consecutive days starting on postoperative day 4. Sham-operated rats underwent identical treatments. TWL was measured before surgery (baseline), before drug treatment (pre-dosing), and 30 min after drug administration (post-dosing) on the morning of postoperative day 6, 8, 10, 12, and 14.

Mechanical allodynia was determined with a commercial electronic von Frey apparatus (Model 2390; IITC Life Science Inc., Woodland Hills, CA, USA) as described by Mitrirattanakul et al. [49], with minor modifications. Briefly, the rats were placed in a Plexiglas box on a steel mesh floor. The center of the hind paw was stimulated using the von Frey filament applied up to a maximum strength of 55 g or until the point of paw withdrawal. The threshold at which withdrawal occurred was automatically registered. The procedure was performed twice for each hind paw at 10-min intervals. The MWT was calculated as the mean of the 2 thresholds.

The MWT was measured 30 min after the TWL measurement in sham-operated rats and in rats receiving the vehicle, koumine (0.28, 1.4, or 7.0 mg kg^−1^ bw), or gabapentin (40 mg kg^−1^ bw) twice daily by s.c. injection for 7 consecutive days starting on postoperative day 4.

### Immunofluorescence

The rats were assigned to naïve, sham, CCI, CCI with 7 mg kg^−1^ bw koumine, and CCI with 0.28 mg kg^−1^ bw koumine groups. Koumine and the vehicle were administered by s.c. injection at a volume of 0.25 mL/100 g bw twice daily for 7 consecutive days starting from postoperative day 4. The rats were anesthetized by i.p. injection of 400 mg kg^−1^ bw chloral hydrate 1 h after drug administration on the morning of postoperative day 5, 7, 10, or 14. The L5–L6 of the SC were excised for analysis by fluorescence immunohistochemistry as described previously, with minor modifications [21]. Briefly, 100 mL of 0.1 M phosphate buffer (PB, pH 7.4) was perfused transcardially followed by perfusion with 450 mL of 4% formaldehyde prepared in PB (fixative solution). The SC located between L5 and L6 was rapidly dissected and fixed in fixative solution for 24 h. The SC tissue was immersed in 15% sucrose-containing PB for 12 h and was subsequently transferred into 30% sucrose-containing PB for 24 h. The SC tissue was then placed in Tissue-Tek^®^ OCT embedding medium (Sakura, Torrance, CA, USA), and was immediately frozen at −22°C. Coronal sections (16 μm thick) were cut on a Microm HM 525E cryostat (Francheville, France) and were subsequently mounted on glass slides coated with gelatin and chromium potassium sulfate.

The SC sections were preincubated for 1 h with the following sera in preparation for subsequent immunohistochemical experiments: (1) for mono-labeling with anti-3α-HSOR, anti-NeuN, or anti-GFAP, and double-labeling with anti-3α-HSOR and anti-NeuN or anti-GFAP, the SC sections were preincubated with 10% nonimmune goat serum prepared in PB containing 0.3% Triton X-100 (PBT); (2) for mono-labeling with anti-Iba1, and double-labeling with anti-3α-HSOR and anti-Iba1, the SC sections were preincubated with 10% nonimmune donkey serum prepared in PBT.

The immunohistochemical mono-labeling experiments were conducted by incubating the SC sections for 24 h at 4°C with a single antibody (anti-3α-HSOR, 1:500 dilution; anti-NeuN, 1:1,000 dilution; anti-Iba1, 1:500 dilution; anti-GFAP, 1:400 dilution) prepared in PBT. In the immunohistochemical double-labeling experiments, the sections were incubated with anti-3α-HSOR in combination with anti-NeuN, anti-Iba1, or anti-GFAP prepared in PBT at the same dilution ratios used for the mono labeling experiments. After washing for 4 times in phosphate-buffered saline (PBS, 5 min per rinse), the sections were transferred into a solution containing either a single secondary antibody (mono-labeling) or multiple antibodies (double-labeling), and were incubated for 1 h at room temperature. The mono-labeling solutions contained either TRITC-conjugated goat anti-rabbit, FITC-conjugated goat anti-mouse, or FITC-conjugated donkey anti-goat secondary antibody prepared in PBT at a dilution ratio of 1:300. The double-labeling solutions contained a mixture of the appropriate two secondary antibodies. After rinsing 3 times in PBS (5 min per rinse), the sections were mounted with anti-fade mounting medium (Beyotime, Haimen China), and imaged under a fluorescence DMR microscope equipped with a digital camera (IX71-A12FL/PH, Olympus, Tokyo, Japan) connected to a Pentium 4 PC. The images were adjusted using PhotoShop (Version 7; Adobe Systems, Inc., San Jose, CA, USA) and the fluorescence density was analyzed using the Image-Pro Plus software (Version 6.0; Media Cybernetics, Rockville, MD, USA).

### Reverse transcription and real-time PCR

The SC located between L5 and L6 was excised from rats anesthetized by i.p. injection of 400 mg kg^−1^ bw chloral hydrate at 5, 7, 10, or 14 days after sciatic nerve ligature or the sham procedure. Total RNA was extracted from tissue samples using TRIzol reagent (Invitrogen, Carlsbad, CA, USA) according to the manufacturer’s instructions. RNA quality was determined by electrophoresis using ethidium bromide-stained agarose gels, and was further confirmed by an optical density (OD) absorption ratio (OD 260 nm/OD 280 nm) >1.7. A fixed quantity of total RNA (1 μg) was subjected to reverse transcription (RT) PCR performed at 37°C for 15 min. The reaction for first-strand cDNA contained the following: 5 × PrimeScript^®^ Buffer (4 µL), 50 µM Oligo dT Primer (1 µL), 100 µM random 6 mers (1 µL), PrimeScript^®^ RT Enzyme Mix I (1 µL), and total RNA (1 µg) added to RNase Free dH_2_O in a final volume of 20 μL. Real-time PCR (rt-PCR) experiments were performed using a LightCycler system (Roche Diagnostics GmbH, Mannheim, Germany). The primer sequences were as follows: *3α*-*HSOR* sense: 5′-TTCATTCCTGTACTGGG-3′ and *3α*-*HSOR* antisense: 5′-AGTAGCCTTGATAACTTCAT-3′ [50], glyceraldehyde-3-phosphate dehydrogenase (*GAPDH*) sense: 5′-ACCACAGTCCATGCCATCAC-3′ (nucleotides 3,069–3,088) and *GAPDH* antisense: 5′-TCCACCACCCTGTTGCTGTA-3′ (nucleotides 3,624–3,605). All primers were chemically synthesized by Sangon Biotech (Shanghai, China). The housekeeping gene, *GAPDH*, was used as an internal control. The rt-PCR experiments were performed in a total volume of 20 μL containing cDNA (2 μL), 10 μM specific primers (1.6 μL), and SYBR Premix Ex Taq (10 μL) containing Taq polymerase, deoxyribonucleotide triphosphate, MgCl_2_, SYBR Green I dye, and dH_2_O (6.4 µL). A sample without the cDNA template (water) was used as a negative control and was run in every assay. Each PCR reaction was performed in triplicate according to the following amplification protocol: a 30-s denaturing step at 95°C, 5-s amplification cycle at 95°C, 5-s amplification cycle at 55°C, and 12-s amplification cycle at 62°C in a total of 45 cycles. The specificity of the rt-RT-PCR products and the absence of non-specific products were confirmed by examining the melting curve. The concentration of *3α*-*HSOR* mRNA in each sample was determined after normalizing the rt-RT-PCR *3α*-*HSOR* product to that of *GAPDH*.

### Enzymatic activity assay of 3α-HSOR

To investigate the enzymatic activity of 3α-HSOR in the L5 to L6 region of the SC following koumine administration, CCI- or sham-operated rats were assigned to vehicle, sham-operated, or koumine (0.28 or 7.0 mg kg^−1^ bw) treatment groups. A naïve group was used as additional control. Vehicle or koumine was administered by s.c. injection twice daily for 7 consecutive days starting on postoperative day 4. On postoperative day 10, the rats were euthanized by decapitation, and the SC L5 to L6 were rapidly excised and stored at −80°C. Enzyme activity assays for 3α-HSOR were performed as described previously [51, 52], with minor modifications. Briefly, 3α-HSOR activity was determined by spectrophotometric measurement (UV-2450, Shimadzu, Kyoto, Japan) of NADPH oxidation at 340 nm and 37°C using a 1.0-cm path length cuvette. The excised SC sections were homogenized in 2 mL of ice-cold 10 mM PB (pH 6.5) containing 0.154 M KCl, 1 mM dithiothreitol, 0.5 mM ethylenediaminetetraacetic acid (EDTA), and 1 μM phenylmethanesulfonyl fluoride (PMSF). The homogenate was centrifuged at 105,000×*g* for 60 min at 4°C in an Eppendorf 5430R ultracentrifuge (Hamburg, Germany). The supernatant (cytosolic) fraction was stored at −80°C until required for the enzymatic activity assay and quantitative analysis. Reductase activity was measured in 100 mM PB (pH 6.5) containing 0.1 mM NADPH, 0.08 mM 5α-DHP (substrate), and enzyme solution (80 μL of the cytosolic fraction) in a total volume of 0.7 mL. The reaction was initiated by addition of the cofactor to the assay mixture, and a blank sample without substrate was included in the measurements. The protein concentrations were determined using the Enhanced BCA Protein Assay Kit (Beyotime Biotech, Haimen, China).

### Statistical analysis

Continuous data were expressed as the mean ± SEM unless otherwise indicated. The TWL and MWT responses were analyzed using two-way repeated measures ANOVA (treatment and time). The significant differences between the groups were determined by Bonferroni post hoc test. The data from the 3α-HSOR catalytic activity and MPA inhibition tests were analyzed using one-way ANOVA with Bonferroni post hoc test. Immunohistochemistry and rtPCR data were analyzed using two-way ANOVA (treatment and time), followed by either Dunnett’s T3 or Bonferroni post hoc test. Differences were considered statistically significant if *P* < 0.05. Statistical analyses were performed with the Statistical Package for the Social Sciences software (SPSS version 13.0; SPSS Inc., Chicago, IL, USA).

## Abbreviations

CCI: chronic constriction injury
DMSO: dimethyl sulfoxide
3α-HSOR: 3α-hydroxysteroid dehydrogenase
EDTA: ethylenediaminetetraacetic acid
GAPDH: glyceraldehyde-3-phosphate dehydrogenase
GFAP: glial fibrillary acid protein
Iba1: ionized calcium binding adaptor molecule 1
L5-L6: lumbar region 5 and 6
MWT: mechanical withdrawal threshold
MPA: medroxyprogesterone acetate
NADPH: nicotinamide adenine dinucleotide phosphate
NeuN: neuronal nuclei
NS: normal saline
NSAIDs: nonsteroidal anti-inflammatory drugs
OD: optical density
PB: phosphate buffer
PBS: phosphate-buffered saline
PBT: PB containing 0.3% Triton X-100
PMSF: phenylmethanesulfonyl fluoride
rt-PCR: real-time polymerase chain reaction
RT-PCR: reverse transcriptase PCR
SC: spinal cord
SNL: spinal nerve ligation
3α, 5α-THP: 3α, 5α-tetrahydroprogesterone
PE: polyethylene
TWL: thermal withdrawal latency
